# Artificial Intelligence in Vascular and Endovascular Surgery: From Prediction to Surgeon-Led Clinical Implementation

**DOI:** 10.3400/avd.ra.26-00128

**Published:** 2026-10-10

**Authors:** Tomohiro Nakajima, Toshiya Nishibe, Yutaka Iba, Junji Nakazawa, Tsuyoshi Shibata, Ayaka Arihara, Hiroki Ishige, Kyosuke Miki

**Affiliations:** 1 Division of Cardiovascular Surgery, Department of Surgery, Sapporo Medical University, Sapporo, Hokkaido, Japan; 2 Faculty of Medical Informatics, Hokkaido Information University, Ebetsu, Hokkaido, Japan

**Keywords:** artificial intelligence, vascular surgery, endovascular surgery, clinical implementation, machine learning

## Abstract

Artificial intelligence (AI) has shown promising performance in vascular imaging, risk prediction, procedural support, surveillance, and education, but its translation into routine surgical care remains limited. This narrative review addresses a focused question: what evidence supports the transition of vascular AI from technical prediction to surgeon-led clinical implementation? A targeted PubMed search through July 24, 2026 identified international and Japan-based studies across the perioperative pathway. Deep-learning systems can segment aortic anatomy, characterize aneurysm or dissection, and support post-EVAR imaging. Machine-learning models may stratify mortality and treatment risk, whereas computer vision, image fusion, wearable monitoring, and large language models extend potential use into procedures, follow-up, and education. Japanese reports demonstrate locally relevant innovation in aortic imaging, EVAR prognosis, postoperative monitoring, and specialty education; however, most evidence remains retrospective, single-center, or developmental. Selected surgeon-led workflows, including quantitative spinal cord imaging after aortic repair, are presented with explicit data provenance as illustrative demonstrations, not proof of clinical efficacy. Progress requires external validation, workflow and human-factors evaluation, prospective impact studies, transparent reporting, and post-deployment monitoring. AI should augment vascular specialists, with surgeons retaining responsibility for clinical questions, verification, interpretation, and patient care.

## Introduction: The Implementation Gap

Artificial intelligence (AI) is advancing rapidly across surgery, yet the central problem is no longer whether an algorithm can produce a prediction. The clinically important question is whether that prediction improves a clinical decision, surgical procedure, follow-up strategy, or patient outcome. Reviews of vascular and cardiac surgery consistently show strong technical results but limited external validation and little evidence of routine clinical impact.^1–5)^

Vascular and endovascular surgery are well suited to computational support because they combine cross-sectional imaging, measurable anatomy, device-based procedures, longitudinal surveillance, and high-consequence events. However, these same characteristics also create hazards: models may learn scanner-, device-, or institution-specific patterns and fail when anatomy, protocols, or populations change. The surgeon must therefore remain the clinical integrator rather than a passive recipient of algorithmic output.

This narrative review addresses 1 focused question: what evidence supports the transition of AI in vascular and endovascular surgery from technical prediction to surgeon-led clinical implementation? Evidence is organized along the care pathway, a Japan-based literature subset is examined, and selected local workflows are used only to illustrate how surgeons can supervise implementation.

## Literature Search Strategy and Narrative-Review Framework

A targeted PubMed/MEDLINE search was performed by 1 reviewer through July 24, 2026. Search terms combined “artificial intelligence,” “machine learning,” “deep learning,” “large language model,” “vascular surgery,” “cardiovascular surgery,” “endovascular,” “aortic aneurysm,” “EVAR,” “aortic dissection,” “carotid,” “peripheral artery disease,” “endoleak,” “Japan,” and related terms. Reference lists of relevant reviews and reporting guidelines were also examined. Priority was given to original clinical studies, external or multicenter validation, prospective evaluation, clinically meaningful outcomes, and recent work that materially changed the field.

This was a narrative, not a systematic, review. Duplicate independent screening, formal risk-of-bias assessment, protocol registration, and meta-analysis were not performed. The manuscript was structured with reference to SANRA: the importance and aim of the review are explicit; the search is described; claims are referenced; evidence level and limitations are discussed; and clinically relevant endpoints are distinguished from technical metrics.^6)^ All cited references are indexed in PubMed.

**Table 1** summarizes the key studies discussed below by stage of the care pathway, with study design, sample size, endpoint, principal performance metric, and type of validation. The pattern it reveals is consistent: of the 22 primary studies listed, only 4 were validated at an institution other than the development site, 1 reported temporal validation only, 1 was prospective in design and 1 included a prospective external cohort, and only 2 reported a patient-relevant endpoint such as contrast volume or radiation exposure rather than a discrimination or segmentation metric.

**Table 1:** Key artificial-intelligence studies in vascular and endovascular surgery, by stage of the care pathway

Ref	Design	Sample size	Endpoint	Key performance	Validation
Preoperative imaging and disease characterization					
7	Retrospective, deep-learning segmentation	500 patients; 2495 3-D+t ultrasound images	AAA segmentation quality	Poorly rated segmentations 8% vs. 18% (conventional)	Independent test group; CT and reader comparison
8	Retrospective, 2 centers, multimodal	561 patients; 14252 CT images	Rapid progression (>2.5 mm/year)	AUC 0.807 vs. 0.716 (clinical), 0.715 (geometric)	Internal
9	Retrospective, matched case-control	80 ruptured; 80 matched controls	AAA rupture	AUC NR; authors note possible overfitting	Internal only
10	Longitudinal, geometric deep learning	113 CTA/24 patients	Local growth; repair eligibility at 2 years	Median diameter error 1.18 mm; accuracy 0.93	External (25 CTA/7 patients, other hospital)
11	Retrospective, propensity-matched	263 patients (temporal test 33)	Impending rupture, symptomatic AAA	AUC 0.898; 0.880 on temporal test; clinical rule 0.751	Temporal
12	Retrospective, 3 centers	861 cases	Aortic dissection detection	AUC 0.98 (noncontrast CT), 0.99 (CTA)	Multicentre test sets
13	Retrospective, single center	498 patients	Stanford type A vs. B vs. normal	Sensitivity/specificity 0.969/0.982 (type A)	Independent test set (316 patients)
14	Retrospective, deep-learning segmentation	390 images/376 individuals	Carotid wall segmentation; stenosis	DSC 95.2%/96.3%; 92.4% externally	External (122 images/115 individuals)
15	Retrospective development, prospective external, multicenter	923 patients; 143 external	Vulnerable plaque and acute ischemic stroke	AUC 0.917; 0.866 externally; 0.719 ultrasound alone	External, prospective, 3 centres
Risk prediction and perioperative decisions					
16	Systematic review and critical appraisal	18 studies	AAA growth or rupture	AUC 0.79–0.93 (growth); 0.75–0.91 (rupture)	External validation uncommon among included studies
17	Retrospective registry (2011–2021)	6601 patients; 470 events (7.1%)	30-day major adverse limb event or death	AUC 0.94 (gradient boosting) vs. 0.74 (logistic regression)	Internal (70/30 split, 10-fold CV)
18	Retrospective, single center, decision tree	169 patients	3-year mortality after EVAR	Accuracy 68.7%; sensitivity 79.0%	Internal
19	Retrospective, single center, several algorithms	142 patients	5-year all-cause mortality after EVAR	Training AUC 0.80; cross-validated 0.77	Internal (5-fold CV); external validation recommended by authors
20	Retrospective, single center, random forest	176 patients (169 analyzed)	3-year survival after EVAR	Training AUC 0.91; cross-validated 0.76	Internal (5-fold CV)
Intraoperative guidance and endovascular workflow					
21	Retrospective, single center	500 images/31 FEVAR procedures	Fenestration marker localization; graft segmentation	Median error 0.16 mm; DSC 96.6% (graft)	Internal, patient-wise split
22	Retrospective comparative, single center	53 without vs. 63 with fusion imaging	Contrast volume; radiation; procedure time	Contrast 105 to 83 mL (p = 0.005); radiation reduced (p <0.001); times not significant	Clinical comparison; no model validation
Postoperative surveillance and monitoring					
23	Retrospective, single center	334 CTA examinations/191 patients	Endoleak detection; diameter and volume	AUC 0.94; endoleak volume DSC 0.53	Internal (5-fold CV); external validation stated as required
24	Retrospective evaluation of a commercial tool	94 patients	Endoleak detection on dual-energy CTA	Accuracy 78.7%; AUC 0.77; failed on monoenergetic images	Independent, against 2-reader consensus
Japan-based studies					
25	Deep-learning image synthesis	NR	Noncontrast to contrast CT synthesis; AAA detection	F score 0.58 (slice); 0.85 (case)	NR
26	Retrospective, ethics approved	112 CT scans (57 type A, 55 type B)	Segmental volumetry in aortic dissection	True-lumen DSC 0.877 to 0.945; HD95 16.9 to 4.4 mm	Internal; limits of agreement about +/−40%
27	Retrospective, single center	4306 images/87 patients	Maskless 2-D subtraction angiography	PSNR 43.1 dB; SSIM 0.98	Internal (motion-free and motion-artifact test sets)
28	Prospective, single center	44 patients	Post-EVAR sac diameter; radiation dose	Mean difference −0.37 mm; CTDIvol 6.9 to 1.0 mGy	Prospective within-patient comparison
29	Cross-sectional pilot, single center	56 dissection vs. 444 controls	Angioscopic features associated with dissection	Random forest with LASSO; intramural blood most influential	None; multicentre validation recommended by authors

Values were taken from the primary publication of each cited study.

NR, not reported; AAA, abdominal aortic aneurysm; AUC, area under the receiver operating characteristic curve; CTA, computed tomography angiography; DSC, dice similarity coefficient; CV, cross-validation; EVAR, endovascular aneurysm repair; PSNR, peak signal-to-noise ratio; SSIM, structural similarity index measure; CTDIvol, volume computed tomography dose index; LASSO, least absolute shrinkage and selection operator

## Preoperative Imaging and Disease Characterization

Aortic aneurysm segmentation and risk characterization. Automated segmentation can reduce the manual work required to quantify aneurysm diameter, volume, thrombus, and 3-dimensional morphology. In a time-resolved 3-dimensional ultrasound study, deep-learning models improved segmentation metrics and halved the proportion of poorly rated segmentations relative to a conventional method (**Table 1**).^7)^ Such automation may make volumetric and biomechanical surveillance more reproducible, but segmentation accuracy must be evaluated across scanners, acquisition protocols, and challenging postoperative anatomy.

AI models are also being used to move beyond maximum diameter as the sole marker of abdominal aortic aneurysm (AAA) risk. A multimodal model integrating CT, geometric, and clinical features predicted rapid progression better than models based on clinical or geometric variables alone (**Table 1**).^8)^ Other studies have examined calcification distribution, local surface growth, and symptomatic rupture using imaging plus clinical data.^9–11)^ These approaches are scientifically attractive because they capture heterogeneity that diameter cannot, but their clinical value depends on prospective confirmation that predictions change surveillance or repair decisions without causing inappropriate intervention.

Aortic dissection. Deep learning has shown high diagnostic performance for detection and classification of aortic dissection on CT. A multicenter bimodal model processing either noncontrast CT or CT angiography reported high discrimination for both inputs (**Table 1**).^12)^ An independent CTA study reported high sensitivity and specificity for distinguishing Stanford type A, type B, and normal examinations.^13)^ These systems may support triage and prioritization, particularly where specialist interpretation is delayed; however, the consequences of false negatives are severe, and performance must be tested in unselected emergency cohorts with motion artifacts, postoperative grafts, and alternative acute aortic syndromes.

Carotid disease. Ultrasound-based AI can segment the arterial wall and plaque, quantify stenosis, and extract features associated with vulnerability. A deep-learning system for common carotid ultrasound was tested in an external cohort and achieved high Dice coefficients for lumen-intima and media-adventitia borders.^14)^ A later multicenter study combining B-mode and contrast-enhanced ultrasound features reported an area under the receiver operating characteristic curve (AUC) of 0.866 in external testing for plaques associated with acute ischemic stroke.^15)^ Although these results are encouraging, treatment decisions should not be based on opaque plaque classifications until incremental value over established clinical, anatomic, and physiologic assessment is demonstrated.

## Risk Prediction and Perioperative Decisions

Prediction models seek to combine demographics, comorbidities, laboratory data, anatomy, and procedural variables into individualized estimates. In a critical appraisal of 18 studies predicting AAA growth or rupture, reported AUCs ranged from 0.79 to 0.93 for growth and 0.75 to 0.91 for rupture; external validation was uncommon, and reporting of transparency and fairness was inconsistent.^16)^ This synthesis is more informative than any single high AUC: the main barrier is no longer the creation of a model, but its reliable transport across institutions and the demonstration of its decision benefit.

For peripheral artery disease, a large registry study of endovascular aortoiliac revascularization developed models for 30-day major adverse limb event or death, in which gradient boosting substantially outperformed logistic regression (**Table 1**).^17)^ The large sample and clinically relevant endpoint are strengths, yet the exclusion of open procedures and use of registry variables limit bedside generalization. Calibration, threshold selection, and net benefit should be reported alongside discrimination before models are used to recommend treatment.

Single-center EVAR studies from Japan illustrate both the potential and limitations of institution-specific models. Decision trees, random forests, and comparative machine-learning analyses have been used to predict short- and long-term survival after EVAR.^18–20)^ These studies provide interpretable hypotheses and may support local quality improvement, but small samples and internal validation do not establish transportability. Their appropriate next step is multicenter temporal and geographic validation against transparent regression baselines, followed by evaluation of whether predictions improve shared decision-making. **Table 1** makes the reason explicit: where these studies report both training and cross-validated performance, the validated figure is consistently the lower of the 2.

## Intraoperative Guidance and Endovascular Workflow

Intraoperative AI is developing from image registration and fusion toward real-time computer vision. In fenestrated endovascular aortic repair (FEVAR), a deep-learning system localized fenestration markers to within a fraction of a millimetre and segmented graft contours with high accuracy (**Table 1**).^21)^ This work provides a technical basis for automated orientation and overlay tools, but prospective evaluation is needed to determine whether performance remains stable during device motion, respiration, angulation, overlap, and incomplete deployment.

Existing image-fusion platforms provide an important bridge between technical capability and clinical outcomes. In a retrospective FEVAR cohort, cloud-based intelligent mapping was associated with lower contrast volume and radiation exposure, although operative and fluoroscopy times were not significantly different.^22)^ Future AI systems should be judged by endpoints that matter to patients and teams—contrast exposure, radiation, technical success, complications, cognitive workload, and rescue from error—rather than segmentation accuracy alone.

## Postoperative Surveillance and Longitudinal Monitoring

Longitudinal imaging after endovascular repair is labor-intensive and well suited to automated measurement. A deep-learning pipeline demonstrated feasibility for aneurysm and endograft segmentation, diameter and volume measurement, and endoleak detection (**Table 1**).^23)^ Automated comparison with prior studies could eventually identify sac expansion, migration, component separation, or a new endoleak and prioritize examinations for review.

However, feasibility should not be confused with readiness. An independent evaluation of a commercial endoleak-detection tool reported only moderate accuracy with a clinically important number of misdiagnoses, and the tool failed to process virtual monoenergetic reconstructions altogether (**Table 1**).^24)^ A comparable dissociation appears within a single surveillance model, which detected endoleak with high discrimination yet segmented endoleak volume poorly (**Table 1**).^23)^ Surveillance AI must therefore report failure rates, unsupported image types, and uncertainty. A safe deployment model is likely to be human-in-the-loop triage with automatic quality control rather than autonomous exclusion of complications.

## Evidence Emerging from Japan

Japan-based evidence is still limited in volume but now spans several clinically relevant domains. For aortic imaging, Japanese groups have reported aorta-aware synthesis of contrast-enhanced CT from noncontrast data, maskless subtraction angiography, and AI-based segmental volumetry for aortic dissection.^25–27)^ A prospective Japanese study showed that ultralow-dose CT with deep-learning reconstruction could measure post-EVAR sac diameter with markedly reduced radiation exposure, although image quality was lower than with standard-dose CT.^28)^ An exploratory angioscopy study used machine learning to identify aortic wall features associated with dissection and appropriately called for multicenter validation.^29)^

In outcome prediction, Japanese EVAR cohorts have used decision trees, random forests, support vector machines, and gradient boosting to estimate short- and long-term survival.^18–20)^ A focused review has summarized the role of machine learning in AAA prognosis after EVAR.^30)^ These studies demonstrate feasibility and generate clinically plausible variables, but they share the major limitations of small samples, single-center derivation, and internal validation. They should be viewed as hypothesis-generating models that require temporal and geographic validation before individual treatment decisions.

Japanese cardiovascular-surgical applications also extend beyond the aorta. Wearable photoplethysmography and machine learning have been developed to detect atrial fibrillation after cardiac surgery, while a recent evaluation of 8 large language models on the Japanese cardiovascular surgery board examination showed strong performance by leading models but persistent weakness on image-based questions.^31,32)^ Together, the domestic literature supports a pragmatic strategy: develop around a defined clinical need, compare against current care, report failures as well as successes, and validate across institutions.

## Large Language Models and Surgical Education

Large language models (LLMs) can summarize documents, draft explanations, generate teaching questions, and translate technical information into patient-facing language. Their strong performance on Japanese national medical licensing examinations demonstrates broad medical knowledge but does not establish clinical reliability, and LLM-generated teaching material is useful only when faculty verify objectives, answers, and references.^33–35)^

Evidence in vascular-related communication is emerging. Vascular surgeons have rated several LLMs as potentially useful for explaining radiofrequency ablation for varicose veins, while remaining cautious about recommending them directly to patients.^36)^ In venous thromboembolism, physician raters judged some LLM responses comparable to or better than expert responses for selected education and decision-support questions.^37)^ These controlled experiments do not justify unsupervised use. Model versions change, prompts influence answers, citations may be fabricated, and performance on complex or atypical cases can deteriorate.

## Selected Surgeon-Led Implementation Demonstrations

Selected workflows from Sapporo Medical University and collaborators are included only as illustrative examples of surgeon-led implementation and do not constitute an independent clinical evidence set. **Figure 1** contains AI-assisted surgical schemas generated from textual anatomical and procedural descriptions and does not contain patient-derived imaging. **Figure 2** is an aggregate statistical visualization generated from a de-identified emergency-service dataset. **Figure 3** combines 2 exploratory biomechanical analyses derived from the same de-identified pre-rupture computed tomography angiography (CTA) examination. **Figure 4** shows 2 separate anonymized spinal MRI datasets processed with open-source software.

**Fig. 1:**
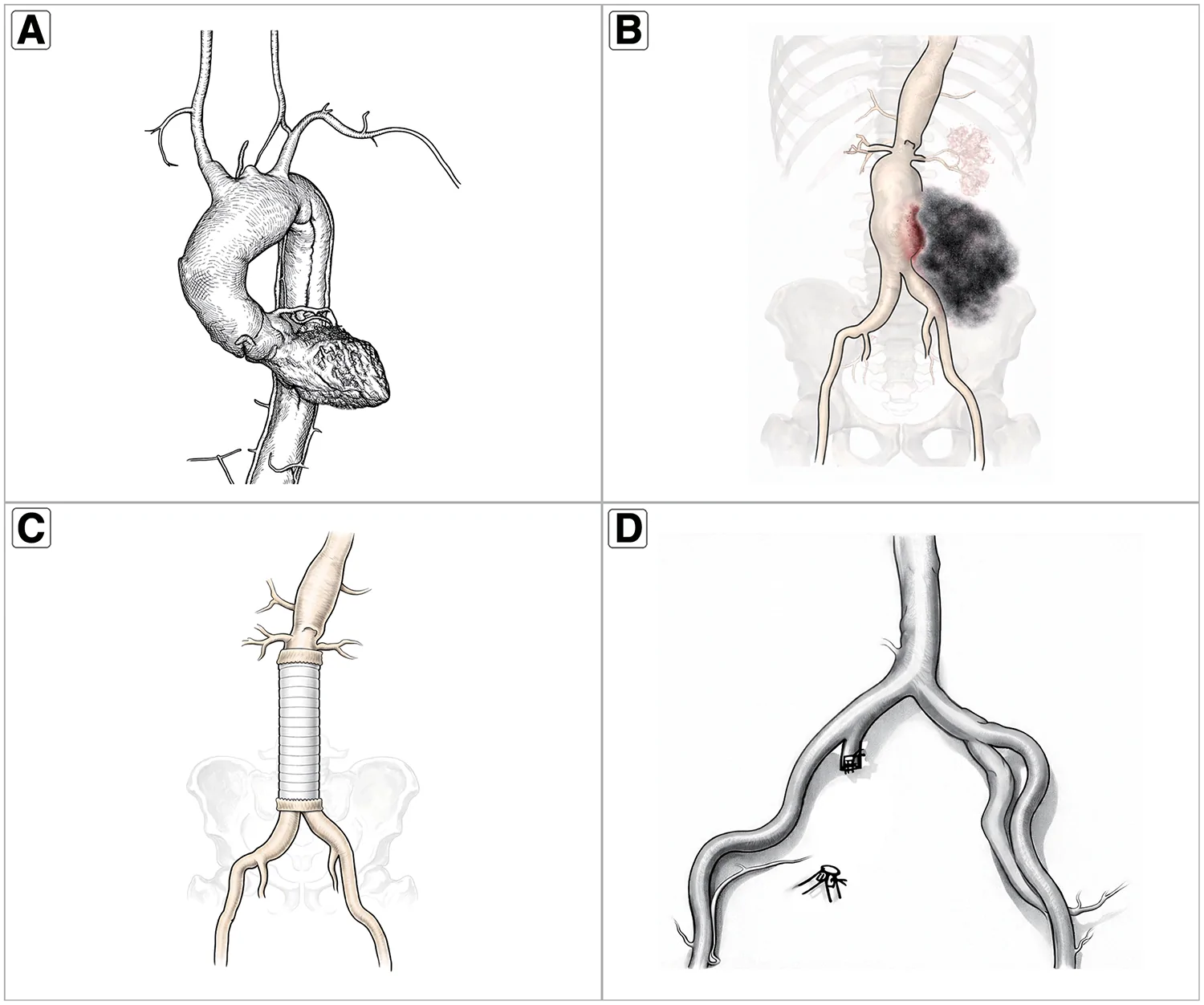
AI-assisted surgical schemas. (**A**) Line-art representation of the heart, ascending aorta, aortic arch, and supra-aortic branches. (**B**) Ruptured abdominal aortic aneurysm with a retroperitoneal hematoma. (**C**) Straight-graft replacement from the infrarenal aorta to the aortic bifurcation. (**D**) Aortoiliac bifurcation and operative landmarks rendered as a monochrome schema. These examples illustrate how natural-language descriptions can be converted into anatomy- and procedure-focused illustrations. All anatomical and procedural details require surgeon review before publication.

**Fig. 2:**
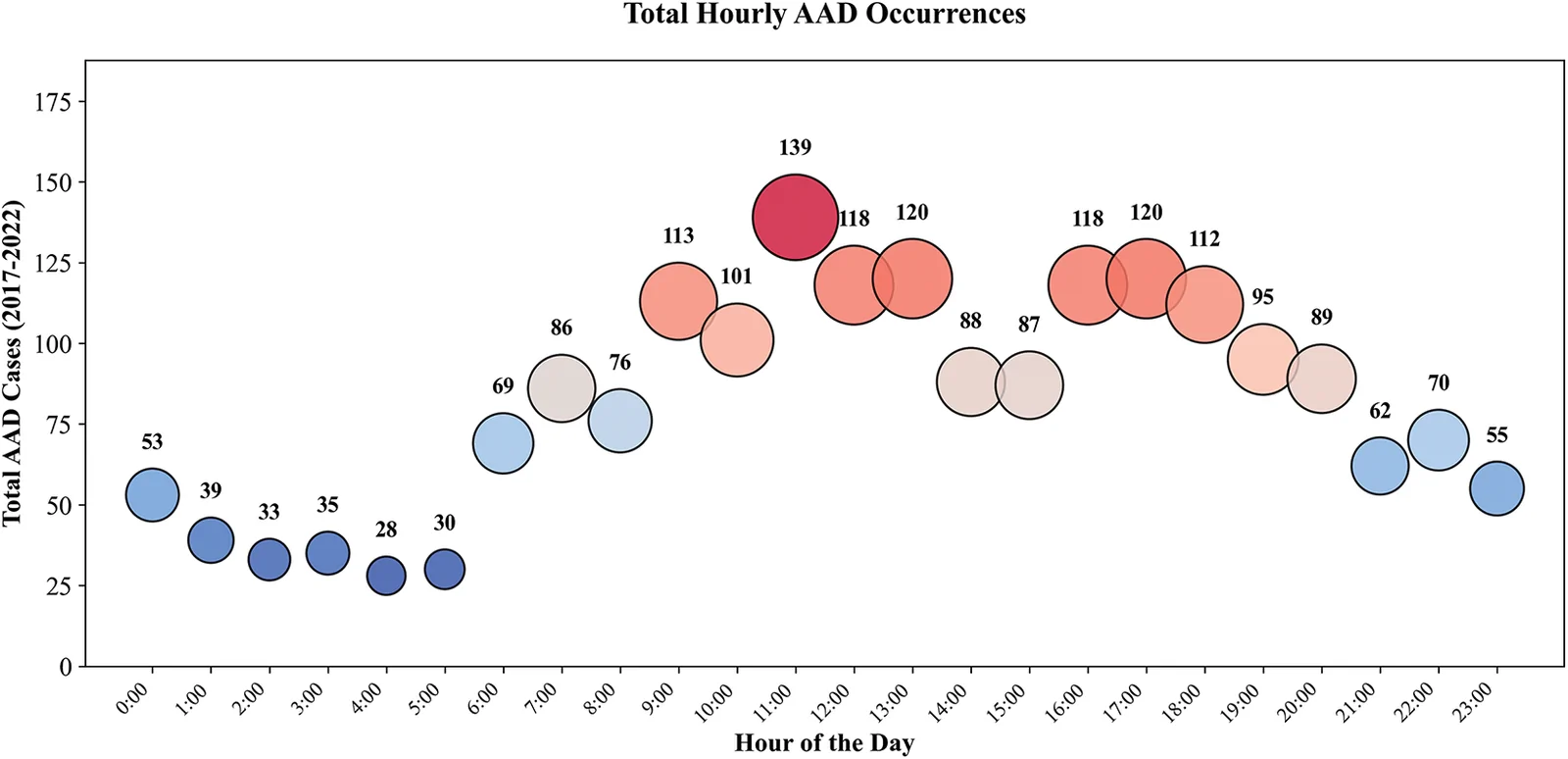
AI-assisted statistical visualization based on aggregate emergency-service data. The source dataset comprised 1936 de-identified acute aortic dissection event records in the Sapporo emergency-service catchment area from January 1, 2017 through December 31, 2022. Bubble position represents the hour of the emergency-call awareness timestamp, which was used as a proxy for onset; bubble size, color, and numeric labels encode the aggregated hourly count. The largest count occurred at 11:00 (139 events). Data processing and plotting were performed in Python/matplotlib with generative-AI assistance for code drafting and visual refinement. No individual-level data are displayed. This descriptive figure does not establish a circadian association.

**Fig. 3:**
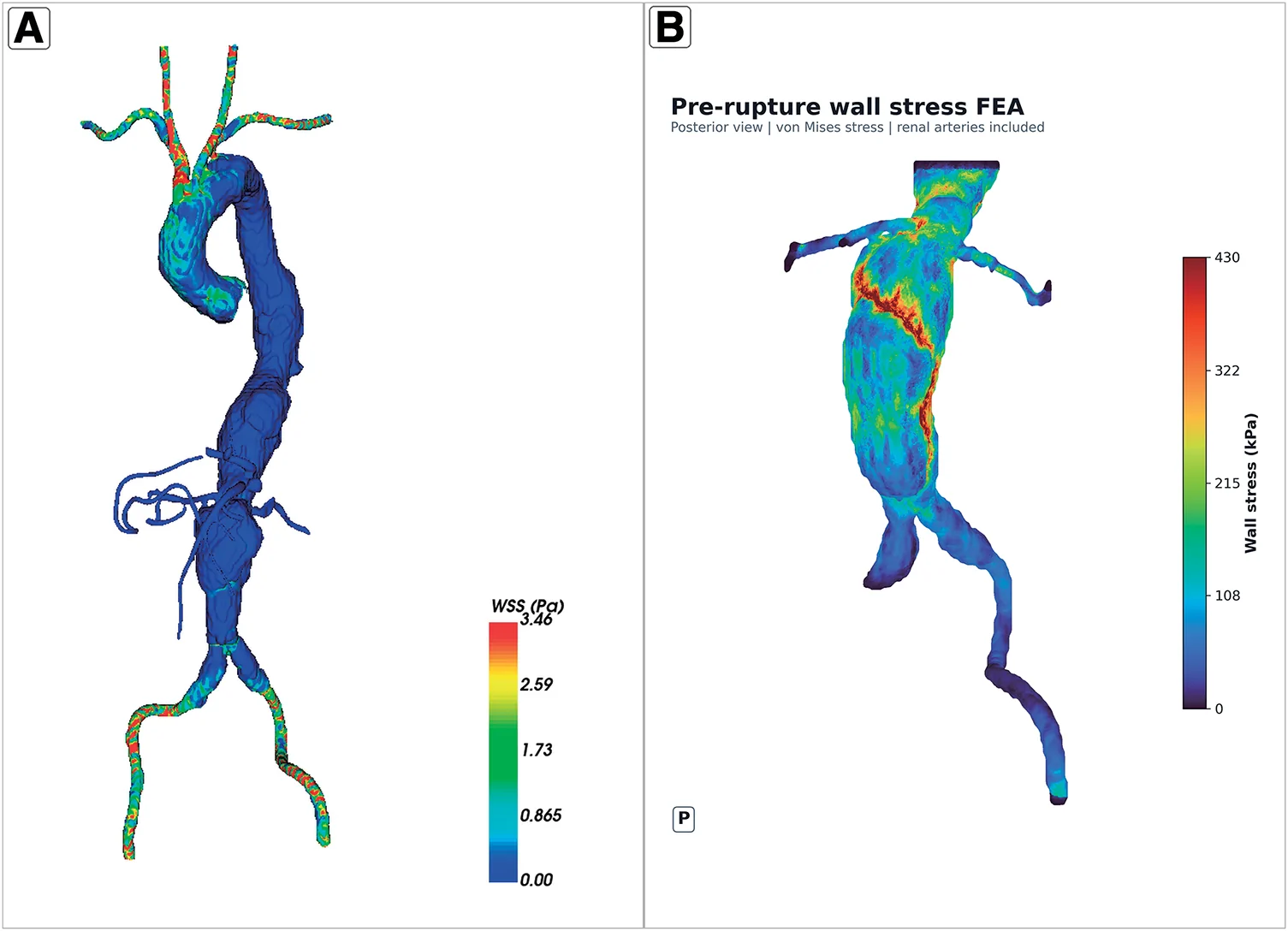
AI-assisted computational biomechanical analysis derived from the same de-identified pre-rupture CTA examination of 1 patient with an infrarenal abdominal aortic aneurysm. (**A**) Posterior-view wall shear stress (WSS; Pa) from a steady, rigid-wall OpenFOAM computational fluid-dynamics model. The aortic geometry was patient-derived, but the flow and boundary assumptions were generic; the result is a qualitative workflow demonstration, not a patient-specific physiological measurement. (**B**) Posterior-view von Mises wall stress (kPa; display range 0–430 kPa) from a separate, renal-inclusive exploratory finite-element model. Generative AI assisted code drafting and debugging; geometry, mesh quality, boundary conditions, material assumptions, convergence, and clinical interpretation remained subject to surgical-team review. WSS and FEA-derived wall stress are distinct quantities. Written informed consent was obtained for publication of clinical details and imaging.

**Fig. 4:**
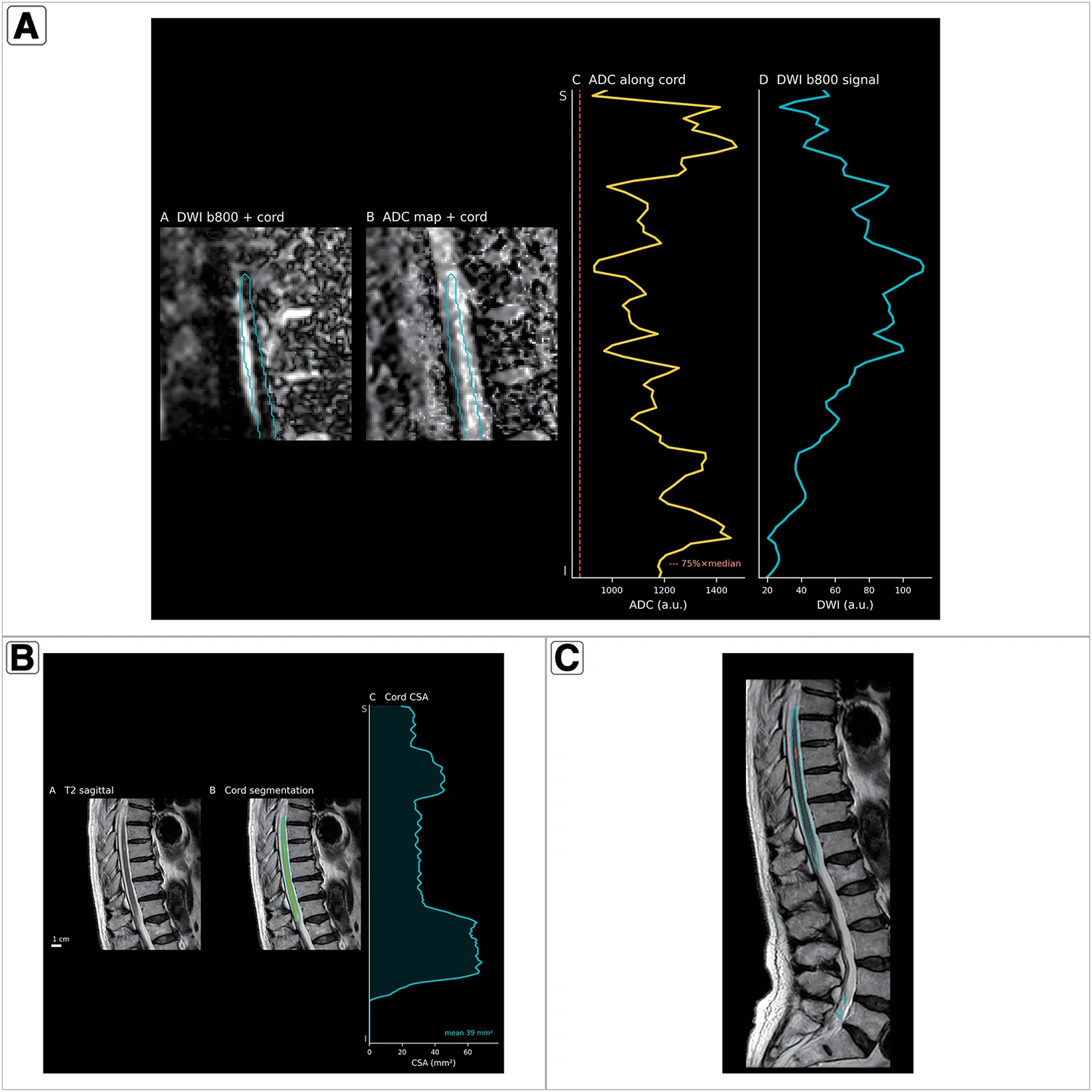
AI-assisted spinal cord MRI analysis using Spinal Cord Toolbox (SCT) version 7.3. The panels were generated from 2 separate anonymized institutional MRI datasets acquired during evaluation of paraplegia after thoracoabdominal aortic repair. Panel (**A**) shows diffusion-weighted imaging at b = 800 s/mm^2^ and an apparent diffusion coefficient (ADC) map, cord-mask overlays, and cranio-caudal ADC and DWI signal profiles. The red line denotes an exploratory display threshold at 75% of the median ADC and is not a validated diagnostic cutoff. Panels (**B**) and (**C**) show a different sagittal T2-weighted MRI dataset: the source image, deep-learning cord segmentation, cord cross-sectional-area profile, and overlays of the cord mask (cyan) and lesion segmentation (red). Automated outputs were visually reviewed. Lesion detection, signal thresholds, and morphometric outputs are workflow demonstrations rather than standalone diagnostic criteria.

For **Fig. 2**, only aggregated hourly counts from a de-identified emergency-service dataset are displayed. The emergency-call awareness timestamp served as a proxy for onset, so the plot depicts call-timestamp counts rather than adjudicated biological onset, and no individual-level information is shown.

For **Fig. 3**, both panels derive from the same de-identified pre-rupture CTA examination of 1 patient with an infrarenal abdominal aortic aneurysm. The geometry was patient-derived, but the flow, boundary, and material assumptions were generic; both panels are therefore qualitative workflow demonstrations rather than patient-specific measurements.^38)^

Spinal cord ischemia causing paraplegia is among the most feared complications of thoracoabdominal aortic aneurysm repair and thoracic endovascular aortic repair, and postoperative imaging findings are often subtle and difficult to quantify. The datasets shown in **Fig. 4** were acquired during evaluation of paraplegia after aortic repair, and the workflow illustrates how cord cross-sectional area and diffusion metrics can be extracted reproducibly using open-source tooling. Quantitative cord assessment is therefore directly relevant to vascular practice, although this figure demonstrates only the processing workflow and not diagnostic performance.

For **Fig. 4**, Panel A was generated from an anonymized diffusion-weighted MRI dataset (b = 800 s/mm^2^ and apparent diffusion coefficient), whereas Panels B and C were generated from a different anonymized sagittal T2-weighted MRI dataset. Spinal Cord Toolbox version 7.3^39)^ was used for spinal-cord segmentation and subsequent processing. The red line in Panel A is an exploratory display threshold at 75% of the median apparent diffusion coefficient and is not a validated diagnostic cutoff. Automated outputs were visually reviewed.

The value of these examples lies in traceability. The clinical question, provenance and de-identification of source data, software and version, commands or prompts, intermediate outputs, and final plausibility checks should be retained. None of the 4 figures establishes diagnostic accuracy, treatment benefit, or generalizability; each should be interpreted as an illustrative workflow until evaluated against a prespecified comparator and clinically meaningful endpoint.

## Requirements for Responsible Clinical Translation

External validation and calibration. Random train-test splitting within 1 institution can overestimate generalizability because acquisition protocols, case mix, prevalence, devices, and clinical documentation are shared. Temporal validation tests performance on later patients; geographic validation tests transport to a new institution. Both should report calibration-in-the-large, calibration slope, clinically relevant thresholds, and subgroup performance in addition to AUC.

Clinical comparison and incremental value. AI should be compared with the actual standard of care, not an artificially weak baseline. For AAA, the relevant question is whether a model improves decisions beyond diameter, growth rate, symptoms, sex, anatomy, and clinician assessment. For postoperative surveillance, the comparison is expert longitudinal review, not isolated image classification. Decision-curve analysis and prospective impact studies can determine whether a model produces benefit at plausible intervention thresholds.

Human factors and workflow. An accurate model can fail if alerts arrive at the wrong time, uncertainty is hidden, or users do not understand the intended population. Early clinical evaluation should document who enters data, who receives the output, how disagreements are resolved, what fallback exists during failure, and whether use changes time, workload, or error. DECIDE-AI provides a framework for reporting these early live evaluations.^40)^

Reproducibility and reporting. Prediction-model studies should follow TRIPOD+AI, including clear descriptions of participants, outcomes, missing data, sample-size reasoning, model specification, evaluation, and availability.^41)^ The FUTURE-AI consensus further emphasizes fairness, universality, traceability, usability, robustness, and explainability across the AI lifecycle.^42)^ These principles are directly relevant to vascular surgery, where rare complications, device evolution, and center-specific practice can cause dataset shift.

Regulatory approval is not equivalent to transparent evidence. A review of AI-based radiology software approved by the Japanese Pharmaceuticals and Medical Devices Agency identified limitations in publicly available information on data composition, validation, and performance.^43)^ Institutions should therefore examine intended use, model version, input requirements, excluded populations, performance by subgroup, cybersecurity, and update policy before adoption.

Generative AI introduces additional risks because a fluent response can be wrong. Hallucinated references, incorrect doses, and adversarially induced errors have been demonstrated across clinical models.^44)^ For vascular care, high-risk outputs include recommendations about emergency triage, anticoagulation, contrast use, device sizing, operative strategy, and postoperative complications. Such outputs require verification against source imaging, records, guidelines, and human expertise; they should never be accepted solely because the prose appears confident.

Privacy and accountability must be designed into the workflow. Minimum-necessary data, de-identification, access control, audit logs, approved storage, and clear retention policies are essential. Local governance should define whether patient data may enter external models and whether model providers may retain or reuse inputs. Responsibility for diagnosis and treatment remains with the licensed clinical team, supported—not displaced—by the algorithm.

The next phase should prioritize multicenter, prospectively registered studies with locked models and clinically meaningful comparators, validated across Asian as well as Western populations, emergency and elective settings, and different devices and scanners. Such studies should ask whether AI changes decisions, reduces time or radiation, detects deterioration earlier, or prevents complications, and should report calibration, decision thresholds, subgroup performance, and failure cases. Silent prospective evaluation can identify dataset shift before outputs influence care, and monitoring must continue after deployment because devices, protocols, and populations evolve. Collaborative registries and shared evaluation protocols may be more valuable than repeated small single-center model development.

## Conclusions

The most defensible role for AI in vascular and endovascular surgery is not autonomous decision-making but surgeon-supervised augmentation of defined clinical tasks. International and Japanese studies show useful signals in imaging, prediction, procedural support, surveillance, monitoring, and education; however, technical accuracy still exceeds evidence of clinical benefit. The next phase should prioritize multicenter validation, prospective workflow studies, patient-relevant endpoints, transparent reporting, and continuous monitoring. A surgeon-led implementation model can preserve accountability while converting computational innovation into safer and more reproducible vascular care.

## Declarations

### Data availability

The individual-level source data underlying **Figs. 2**–**4** are not publicly available because they are subject to privacy, consent, and institutional-governance restrictions. Aggregate information and analysis details are reported in the text and legends. Any additional sharing would require institutional authorization and compliance with applicable data-use conditions.
